# Supplementary material for: Diagnostic Errors in the Acutely Dizzy Patient—Lessons Learned
Source: Brain Sci. 2025 Jan 9;15(1):55. doi: 10.3390/brainsci15010055 (PMC11764146; doi:10.3390/brainsci15010055)
Supplement: Supplementary file 1 [file brainsci-15-00055-s001.zip › brainsci-3397394-supplementary.pdf]

<u>Supplementary material</u>

Supplementary figures and tables for algorithms discussed in the manuscript

Figure S1 - STANDING algorithm.

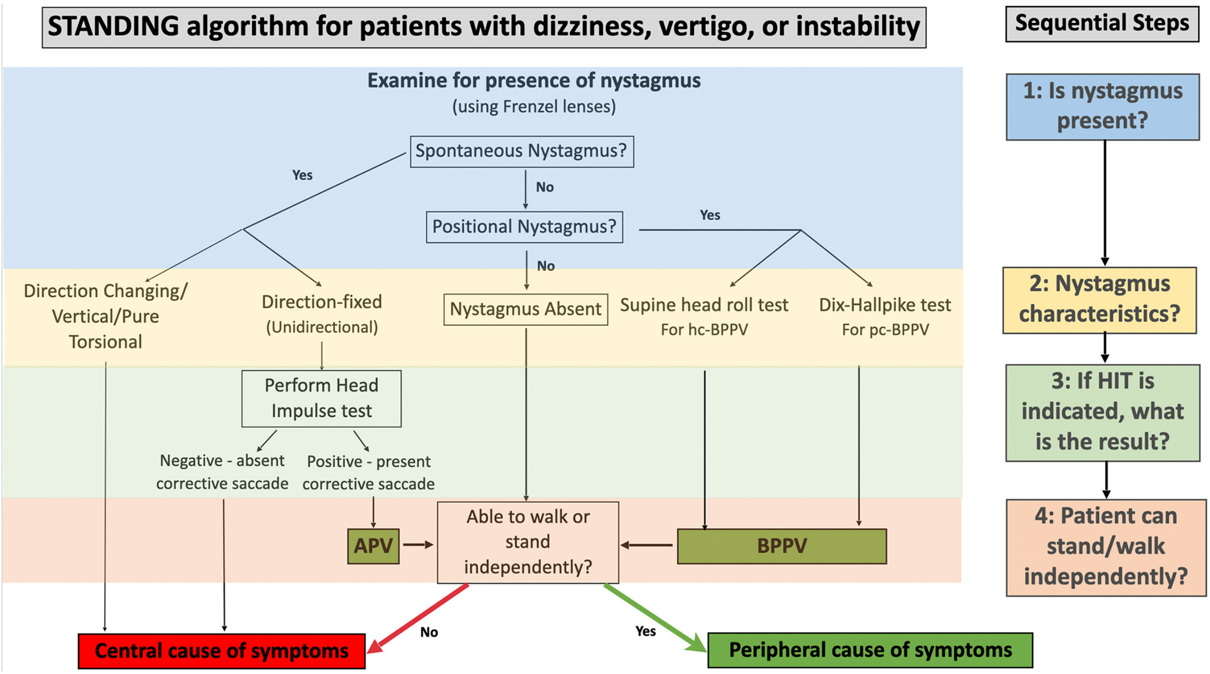

STANDING Algorithm. APV, acute peripheral vestibulopathy (usually vestibular neuritis); BPPV, benign paroxysmal positional vertigo; hc-BPPV, horizontal canal BPPV; HIT, head impulse test; pc-BPPV, posterior canal BPPV. Figure 6 is adapted from Vanni et al. [1,2]

Figure reprinted from [3] with permission from the publisher (John Wiley and Sons)

Figure S2 – Summary receiver operating characteristic curve analysis for various bedside tests compared to brain imaging and clinical scores (retrieved from [4])

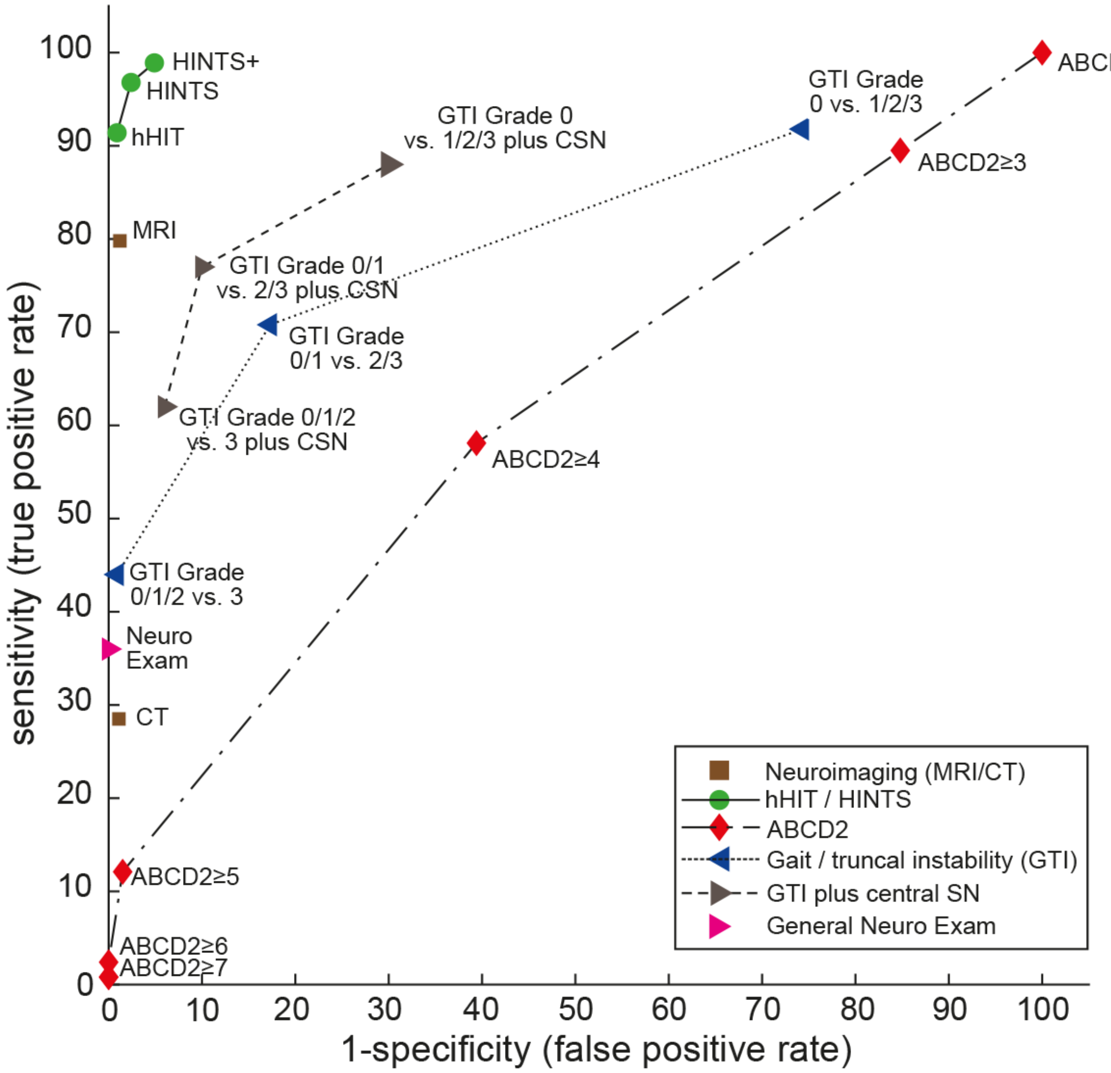

Summary receiver operating characteristic (SROC) curve analysis for the graded gait/truncal instability (GTI) ratings (with or without additional central-type spontaneous/gaze-evoked nystagmus compared to the "HINTS (Head Impulse, Nystagmus, Test of Skew) family", neuroimaging (computed tomography [CT] or magnetic resonance imaging with diffusion-weighted sequences [MRI-DWI], values used as published by Shah and colleagues [5]), general neurologic exam and vascular risk stratification by ABCD2 (age, blood pressure, clinical features, duration of symptoms, diabetes) score (data from a single study [6]) for detecting stroke in patients presenting the acute vestibular syndrome (modified after [7]).
SROC curves are shown for six different diagnostic approaches to diagnosing stroke in the acute vestibular syndrome. A perfect test or decision rule has threshold cutoffs in the upper left corner (100% sensitivity, 100% specificity) and an area under the curve (AUC) of 1.0. Note that the gait/truncal instability ratings outperform the ABCD2 score and the general neurologic exam but are inferior compared to the HINTS family of eye movement tests. This is also true when adding central-type spontaneous nystagmus to the GTI rating. Both HINTS and HINTS plus (HINTS plus new hearing loss detected by finger rubbing or similar) demonstrate a higher diagnostic accuracy for ruling out stroke than MRI including DWI.
Abbreviations: hHIT=horizontal head-impulse test

H.I.N.T.S. plus bedside testing battery (modified after [8])

Table S1: *H.I.N.T.S. plus bedside testing battery**

Test performed	Property evaluated	How to perform this test	Pointing to a peripheral cause	Pointing to a central cause	Comments
**Horizontal Head-Impulse test (HIT)**	Vestibulo-ocular reflex (VOR)	Fast, low amplitude (10-15°) head rotations to the left/right while the patient is looking at a fixed target in space (e.g., the examiner's nose)	Delayed to one side, pathological catch-up saccade	Normal HIT.	Note that central lesions involving the VOR (e.g. lesions in the root-entry zone or of the vestibular nuclei) may show a "pseudo-peripheral pattern"
**Testing for Nystagmus**	Eccentric gaze-holding on lateral gaze	Fixation of an object (e.g. the tip of a pen) during lateral (eccentric) gaze (~20-30°) for at least 5 sec.	Stable eccentric gaze-holding	Deficient eccentric gaze-holding with centripetal drift and centrifugal nystagmus (i.e., left-beating on left-gaze and right-beating on right-gaze).	Spontaneous, predominantly horizontal nystagmus (i.e., primary gaze nystagmus) can be found in both peripheral and central causes and thus allows no differentiation.
**Alternating cover test ("Test of Skew")**	Vertical alignment of the eyes	Rapid covering then uncovering one eye after the other while the patient is looking at a fixed target in space (e.g., the examiner's nose). The examiner should focus on only one eye.	No vertical deviation of the eyes	Vertical realignment of the uncovered eye (one eye goes up while the other eye goes down). This is why it does not matter which eye the examiner focuses on.	Note that rarely a vertical skew can also be observed in peripheral-vestibular deficits, but is usually of smaller amplitude and short-lived.
**New-onset unilateral hearing loss (fourth sign – "plus sign")**	Hearing	Finger rub on each side	Normal hearing	Hearing loss on the side with the abnormal head-impulse test	Hearing may also be compromised in inner ear disorders such as labyrinthitis or complicated otitis media, emphasizing the need for a dedicated examination of the ear.

* Teaching videos can be found under: http://novel.utah.edu/Newman-Toker/collection.php.

Graded rating of gait and truncal instability (GTI)

**Table S2: graded rating of gait and truncal instability (GTI)**	
Grade of gait inability	Definition
0	Normal gait
1	Mild to moderate imbalance but can walk independently [9] or unable to stand on tandem Romberg with the eyes open at least for 3 seconds [10]
2	Severe imbalance with standing and cannot walk without support [9], or unable to stand on Romberg with eyes open for 3 seconds [10].
3	Inability to stand upright unassisted [9,10] or inability to sit upright unassisted [10].

REFERENCES

1. Vanni, S.; Nazerian, P.; Pecci, R.; Pepe, G.; Pavellini, A.; Casula, C.; de Curtis, E.; Ronchetti, M.; Vannucchi, P.; Bartolucci, M. Timing for nystagmus evaluation by STANDING or HINTS in patients with vertigo/dizziness in the emergency department. *Acad Emerg Med* **2023**, *30*, 592-594, doi:10.1111/acem.14635.

2. Vanni, S.; Pecci, R.; Edlow, J.A.; Nazerian, P.; Santimone, R.; Pepe, G.; Moretti, M.; Pavellini, A.; Caviglioli, C.; Casula, C.; et al. Differential Diagnosis of Vertigo in the Emergency Department: A Prospective Validation Study of the STANDING Algorithm. *Front Neurol* **2017**, *8*, 590, doi:10.3389/fneur.2017.00590.

3. Edlow, J.A.; Carpenter, C.; Akhter, M.; Khoujah, D.; Marcolini, E.; Meurer, W.J.; Morrill, D.; Naples, J.G.; Ohle, R.; Omron, R.; et al. Guidelines for reasonable and appropriate care in the emergency department 3 (GRACE-3): Acute dizziness and vertigo in the emergency department. *Acad Emerg Med* **2023**, *30*, 442-486, doi:10.1111/acem.14728.

4. Martinez, C.; Wang, Z.; Zalazar, G.; Carmona, S.; Kattah, J.; Tarnutzer, A.A. Systematic Review and Meta-Analysis of the Diagnostic Accuracy of a Graded Gait and Truncal Instability Rating in Acutely Dizzy and Ataxic Patients. *Cerebellum* **2024**, doi:10.1007/s12311-024-01718-6.

5. Shah, V.P.; Oliveira, J.E.S.L.; Farah, W.; Seisa, M.; Kara Balla, A.; Christensen, A.; Farah, M.; Hasan, B.; Bellolio, F.; Murad, M.H. Diagnostic accuracy of neuroimaging in emergency department patients with acute vertigo or dizziness: A systematic review and meta-analysis for the Guidelines for Reasonable and Appropriate Care in the Emergency Department. *Acad Emerg Med* **2022**, doi:10.1111/acem.14561.

6. Newman-Toker, D.E.; Kerber, K.A.; Hsieh, Y.H.; Pula, J.H.; Omron, R.; Saber Tehrani, A.S.; Mantokoudis, G.; Hanley, D.F.; Zee, D.S.; Kattah, J.C. HINTS outperforms ABCD2 to screen for stroke in acute continuous vertigo and dizziness. *Acad Emerg Med* **2013**, *20*, 986-996, doi:10.1111/acem.12223.

7. Newman-Toker, D.E.; Curthoys, I.S.; Halmagyi, G.M. Diagnosing Stroke in Acute Vertigo: The HINTS Family of Eye Movement Tests and the Future of the "Eye ECG". *Semin Neurol* **2015**, *35*, 506-521, doi:10.1055/s-0035-1564298.

8. Tarnutzer, A.A.; Dieterich, M. Bedside Examination of the Vestibular and Ocular Motor System in Patients with Acute Vertigo or Dizziness. *Clinical and Translational Neuroscience* **2019**, *3*, 19.

9. Lee, H.; Sohn, S.I.; Cho, Y.W.; Lee, S.R.; Ahn, B.H.; Park, B.R.; Baloh, R.W. Cerebellar infarction presenting isolated vertigo: frequency and vascular topographical patterns. *Neurology* **2006**, *67*, 1178-1183, doi:10.1212/01.wnl.0000238500.02302.b4.

10. Moon, I.S.; Kim, J.S.; Choi, K.D.; Kim, M.J.; Oh, S.Y.; Lee, H.; Lee, H.S.; Park, S.H. Isolated nodular infarction. *Stroke* **2009**, *40*, 487-491, doi:10.1161/STROKEAHA.108.527762.
